# Development of closed–loop supply chain network in terms of corporate social responsibility

**DOI:** 10.1371/journal.pone.0174951

**Published:** 2017-04-06

**Authors:** Ali Pedram, Payam Pedram, Nukman Bin Yusoff, Shahryar Sorooshian

**Affiliations:** 1 Centre for Product Design and Manufacturing, Department of Mechanical Engineering, Faculty of Engineering, University of Malaya, Kuala Lumpur, Malaysia; 2 Department of Information Technology, Faculty of Electronic, Computer and Information Technology, Qazvin Islamic Azad University, Qazvin, Iran; 3 Department of Mechanical Engineering, Faculty of Engineering, University of Malaya, Kuala Lumpur, Malaysia; 4 Faculty of Industrial Management, University of Malaysia Pahang, Gambang Kuantan, Pahang, Malaysia; Southwest University, CHINA

## Abstract

Due to the rise in awareness of environmental issues and the depletion of virgin resources, many firms have attempted to increase the sustainability of their activities. One efficient way to elevate sustainability is the consideration of corporate social responsibility (CSR) by designing a closed loop supply chain (CLSC). This paper has developed a mathematical model to increase corporate social responsibility in terms of job creation. Moreover the model, in addition to increasing total CLSC profit, provides a range of strategic decision solutions for decision makers to select a best action plan for a CLSC. A proposed multi-objective mixed-integer linear programming (MILP) model was solved with non-dominated sorting genetic algorithm II (NSGA-II). Fuzzy set theory was employed to select the best compromise solution from the Pareto-optimal solutions. A numerical example was used to validate the potential application of the proposed model. The results highlight the effect of CSR in the design of CLSC.

## Introduction

Scholars, researchers, and policy makers have given tremendous attention to the importance of sustainable development. Taticchi et al. [1] provided a critical literature review to highlight the current status and future trend of sustainable supply chains in both academia and industries. Although the concept of sustainability has received great attention in the literature of supply chain and operations research, studies on the impact of corporate social responsibility (CSR) on sustainable development are less compared to other topics. Oh and Jeong [2] indicated that a sustainable supply chain is at the early stage and a closed-loop supply chain (CLSC) may be an initiative for major progress.

A CLSC has become an important topic [3] because of increased environmental concern and restrictive regulations introduced by governments. For instance, the European Parliament and the Council have established a regulation on waste electrical and electronic equipment—Directive 2012/19/EU [4]–which asked members of the European Union to make cogent changes in their development, production, and consumption behaviour. The regulation also asked for the reduction of wasteful consumption of natural resources and the prevention of pollution [5]. In many countries government regulation forces firms to take responsibility for their products over the entire life cycle. This has led companies to increasingly deal with product returns. To manage product returns, companies require to build reverse supply chains in addition to the forward supply chains. Consequently, this will lead to increased complexity of the supply chain.

On the other hand, regardless of the complexity of the CLSC, it is acknowledged that firms generate value by integrating forward supply chains with reverse supply chains in a CLSC [6, 7]. For example, many companies, such as HP and Xerox Corporation, have established CLSCs and have achieved substantial cost saving [8–10]. Besides, nowadays corporations recognize that the reputation of their brand and their profit margins are closely related to the offer of environmentally friendly services and products [11]. Economic and environmental benefits gained with the reduction of virgin material consumption by using recovered components and parts [7] have led to extended life cycle of products and parts/components [12]. The CLSC network design is an important decision because it is a strategic, tactical, and operational decision. It requires a decision on the locations, numbers and capacities of network facilities, in addition to the material flow through the network [13]. Moreover, network configuration is a critical decision because while you configure the network, any changes require tremendous costs and time [14].

For many years, cost was the main objective for developing a supply chain as well as CLSC. Although there has been an increase in the level of awareness of environmental and social responsibility, few research studies have considered social issues in designing a CLSC network. This has led to a lack of studies in CSR [15]. Companies in fact need to adopt the idea of CSR by pursuing not only economic performance but also environmental and social targets. A CLSC increases a company’s chance of becoming CSR-oriented, improving economic prospects and enhancing competitive advantage for supply chain participants [16].

One aspect of CSR is an increase of opportunities for employment and provision of economic development for local communities. These are of great concern to governments which encourage companies to become CSR-oriented with incentives such as subsidies and tax reduction. For example, the issue of job creation and economic development of the community is addressed in the Iranian Fifth Development Plan [17]. Therefore, considering employment opportunities alongside economic development can be a great help in mitigating worldwide social sustainability concerns.

Accordingly, the purpose of this study is to design an optimal CLSC network. A mathematical model is developed for optimising end of life products within the concept of the CLSC. And the second objective of the study is to focus on CSR in designing a CLSC network. A bi-objective mathematical model maximises profit and number of jobs created. A CLSC is a comprehensive approach for managing both the forward and reverse supply chains [18]. Therefore, the proposed network model is a direction to provide decision support for practitioners of end of life products by determining the number and location of facilities to be opened in view of economic and social benefit and material flows between these facilities.

The rest of the paper is organised as follows. In section 2, relevant literature is reviewed. In section 3, the problem has been defined and the proposed multi-objective mixed integer linear programming model is explained. The results obtained with the proposed model are presented in section 4 and finally the conclusion and direction for future research are expressed in section 5.

## Literature review

Design of a sustainable supply chain requires the consideration of economic, environmental and social aspects. One of the social aspects is unemployment, which has a great effect on society. Van and Storey [19] have found an association between a rise in the rate of formation of new firms and unemployment reduction. They found that an increase in the formation of new firms leads to job creation. Mota et al. [20] developed a multi-objective model for CLSC in order to minimise cost and environmental impact and maximise social benefit. They consider job creation as a factor for social benefit where increase in job creation leads to an increase in social benefit. Although job creation was one of the objectives they did not consider costs for creating the jobs.

The demand for a multi-objective model has increased recently because the problems in the real world are not those of a single objective. This is shown in the number of publications that were published recently with multi-objectives. It is considered as comprising several objectives, which sometimes conflict with each other. Maximising one leads to minimising the other and vice versa. Cost is the main objective for the designing of the CLSC, including transportation cost [21] and facility fixed opening cost [22]. Indeed, CLSC costs could be classified into three main types: transportation costs, fixed opening costs and processing costs. These types of costs are often used based on the network characteristics. In addition to these costs, several authors have considered other type of costs such as inventory cost [23–26], penalty cost [27], and supplier selection cost [24]. Pishvaee et al. [28] in their model have maximised forward and reverse responsiveness in addition to minimising cost of transportation and fixed opening cost. Demirel et al. [29] have developed a model to maximise selling price. Zarandi et al. [3] have developed a model to maximise service level and minimise production and transportation cost. Amin and Zhang [30] designed a network that maximised importance of supplier and also maximised profit.

Easwaran and Üster [31], by the usage of MILP, have developed an optimal solution for their integrated reverse and forward supply chain network to minimise total cost of facility location, processing and transportation. Their model contains a hybrid-sourcing facility (HSF) which played roles as manufacturer and remanufacturer, a hybrid centre which serves as distributor and/or collection centre, and retailers. They have used Benders’ decomposition method to come up with a model with the numerical data. Kannan et al. [32] developed a comprehensive network that has supplier, production centre, distribution centre, wholesaler, and retailer in the forward supply chain and collection centre, disassembly/recycling centre, and disposal centre in the reverse supply chain. The objective of the model is to minimise the total cost of the network such as transportation, processing, and inventory cost and provide a decision tool regarding material procurement, production, distribution, recycling and disposal. They have used genetic algorithm to develop the model. Moreover, to validate the model they have used the production of lead–acid batteries as a case study.

Amin and Zhang [23] have developed a network that remanufactures a product for a secondary market. They have developed a single product and single period model that maximised profit by deducting processing cost, inventory cost, and opening cost from the selling profit. Shi et al. [33] have considered CLSC with the addition of third-party logistics service providers to the forward supply chain. Sasikumar and Haq [34], in addition to developing a CLSC network, considered a third-party reverse logistics provider (3PRLP) to be responsible for the reverse supply chain. Subramanian et al. [26] considered a CLSC network with multi-product and multi-period. Their network involves a production centre, a distribution centre, a wholesaler, and a retailer in the forward SC and collection centre in the reverse SC. The mathematical model is set to minimise the cost. The model was developed using genetic algorithm and particle swarm with the numerical example.

In fact, most of the realistic supply chain models are complex and require a great number of variables and constraints. Therefore, mathematical optimisation methods such as linear programming (LP) may not be very effective for the solution [35]. Furthermore, the problem is NP-Hard because of complexity and exponential growth of the problem size. Hence, it requires the use of mathematical programming algorithms [36]. Therefore, heuristic and meta-heuristic algorithms are used to solve such problems[35]. Among the meta-heuristic algorithms, non-dominated sorting genetic algorithm-II (NSGA) is used to solve the proposed multi-objective model because the performance of the NSGA-II for multi-objective mathematical optimisation is better than other meta-heuristics such as multi-objective particle swarm optimisations (MOPSO)[35, 37].

The exploration in the CLSC network design problem is varied in the form of solution methodology, network configuration, the number of products and periods. Table 1 demonstrates characteristics of some important studies relevant to this research.

**Table 1:** Characteristics of some relevant CLSC network design studies.

Publication	Commodity		Period		Objective		Objectives	Modelling Approach	Multi objective Solution methodology
	Single	Multiple	Single	Multiple	Single	Multiple			
Talaei et al. [38]	x			x		x	Minimization of total cost Minimizing of total carbon dioxide emission	MILP	ɛ-constraint
Ma et al. [39]		x	x			x	Minimization of total cost Minimization of the environmental cost	MINLP	LP-metrics
Subulan et al. [40]		x		x		x	Maximization of total revenue Minimization of total eco-indicator score	MILP	Goal programming
Subulan et al. [41]		x	x			x	Minimization of total cost Maximization of coverage of collected product	MILP	Goal programming
Mota et al. [42]		x	x			x	Minimization of total cost Minimization of environmental impacts Maximization of social impacts	MILP	ɛ-constraint
Ghayebloo et al. [43]		x	x			x	Maximization of total profit Maximization of total greenness	MILP	ɛ-constraint/ weighted sum
Garg et al. [44]		x	x			x	Maximization of total profit Minimization of number of hired vehicle in forward chain	MINLP	Heuristic method
Das and Rao Posinasetti [45]		x	x			x	Maximization of profit Minimization of total energy spent by supply chain	MILP	Pareto optima solutions/ Goal programming
Ramezani et al. [46]		x		x		x	Maximization of net present value (NPV) Minimization of number of defect received from supplier Minimization of delivery time	MILP	Fuzzy multi objective MILP
Dubey and Gunasekaran [47]	x		x			x	Maximization of profit Minimization of Co2 emission related to transportation	MILP	Goal programming
Our study		x		x		x	Maximization of profit Maximization of number of job	MILP	Meta heuristic (NSGA-II)

Based on a systematic review of CLSC and multi-objective supply chain network design articles, the contribution of this work to the literature are:

1. It is among the pioneer articles which consider a social perspective in designing a CLSC network. Although several research studies have been carried out in the field of CLSC network design, corporate social responsibility in developing a CLSC is a new perspective, which this paper aims to shed light on.
2. The development of a conceptual CLSC model that could be applicable in the electronic and automobile industry.
3. The study use NSGA-II as the method for resolving a proposed CLSC network model
4. To the best of the author’s knowledge, this article is the first paper that has used the fuzzy best compromise solution concept in CLSC to give management insight for selecting a best strategy.

## Problem definition and modelling

**Fig 1:**
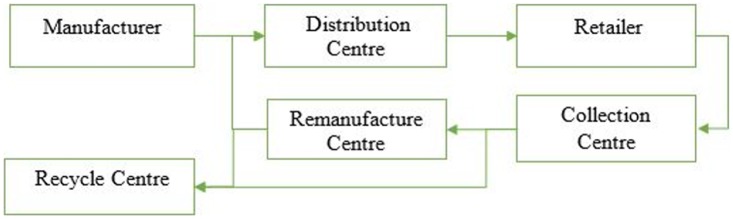
Proposed closed-loop supply chain.

The concerned closed–loop supply chain network is a two-fold, forward and reverse supply chain. The forward supply chain has three tiers including manufacturer, distribution centre and retailer. The reverse flow involves collection centre, remanufacturing centre, and recycling centre. As shown in Fig 1, products shipped through the manufacturers to the distribution centres and retailers are sold to the customers to fulfil the demand. The reverse flow starts with the collection of the used products from the customers. Collection centres, after inspection and quality testing, decide whether to send the used products to the recycling centre or to the remanufacturing centres. At the remanufacturing centres, used products are checked for remanufacturing, refurbishing and repairing. Thus, the used product gains its previous condition and is sent to distribution centre to be sold as a new product at the retailer centre. On the other hand, if the products do not meet the conditions for remanufacturing, they are sent to the recycling centre.

In this study, a CLSC is designed using a mixed integer linear programming model to maximise total profit and number of jobs that are created with a CLSC. The proposed model determines the number of manufacturing centres, distribution centres, retailers, collection centres, remanufacturing centres, and recycling centres. In addition to that, the facility location and the flow between each facility in the particular period is also determined. The structure of the proposed multi-tier, multi-period CLSC for end-of life product is depicted in Fig 1. The discussed network is a generic that can be fitted to any particular industry, such as the electronic industry or the automobile industry. It is assumed that manufacturers have limited capacity of production. Some other assumptions include:

- The capacity of distribution centres, retailers, collection centres, remanufacturing centre and recycling centre are known.
- Demands for new products are provided.
- Cost of manufacturing centre, distribution centre, retailer, collection centre, remanufacturing centre and recycling centre are known.
- Rate of return, recycling and remanufacturing are identified.
- Location of manufacturers, distribution centres, retailers, customers, collection centres, remanufacturing centres, recycle centres are fixed and predefined.

The discussed CLSC network has two objectives as follows: (i) maximising of total profit (ii) maximising job creation. In fact, profit and job creation are two conflicting objectives, in which increase in job creation would cause an increase in cost and reduction of profit.

### Notation and model formulation

We present the mixed-integer linear mathematical model, beginning with the notations.

#### Notations

Indices:

*m*: set of manufacturer m∈M
*dc*: set of distribution centre dc∈DC
*r*: set of retailer r∈R
*cc*: set of collect centre cc∈ CC
*rm*: set of remanufacturing centre rm∈RM
*rc*: set of recycling centre rc∈RC
*u*: set of product type u∈U
*p*: set of period p∈P

Parameters:

*om*_*m*_: fixed opening cost of manufacturer m ∈ M
*odc*_*dc*_: fixed opening cost of distribution centre dc ∈ DC
*or*_*r*_: fixed opening cost of retailer r ∈ R
*occ*_*cc*_: fixed opening cost of collection centre cc ∈ CC
*orm*_*rm*_: fixed opening cost of remanufacturing centre rm ∈ RM
*orc*_*rc*_: fixed opening cost of recycling centre rc ∈ RC
*km*_*m*_: production capacity of manufacturer m ∈ M
*kdc*_*dc*_: holding capacity of distribution centre dc ∈ DC
*kr*_*r*_: holding capacity of retailer r ∈R
*kcc*_*cc*_: holding capacity of collection centre cc∈ CC
*krm*_*rm*_: holding capacity of remanufacturing centre rm ∈ RM
*krc*_*rc*_: holding capacity of recycling centre rc ∈ RC
*jm*_*m*_: Number of jobs create with manufacturer m ∈ M
*jdc*_*dc*_: Number of jobs create with distribution centre dc ∈ DC
*jr*_*r*_: Number of jobs create with retailer r ∈ R
*jcc*_*cc*_: Number of jobs create with collection centre cc ∈ CC
*jrm*_*rm*_: Number of jobs create with remanufacturing centre rm ∈ RM
*jrc*_*rc*_: Number of jobs create with recycling centre rc ∈ RC
*pr*_r,*u*,*p*_: penalty cost for non-satisfied demand for product u ∈ U in period p ∈ P
*p*_*u*_: selling price for product u ∈ U
*dr*_r,*u*,*p*_: demand of retailer r ∈ R for product u ∈ U in period p ∈ P
*sm*_*m*_: cost of creation of jobs in manufacturer m ∈ M
*sdc*_*dc*_: cost of creation of jobs in distribution centre dc ∈ DC
*sr*_*r*_: cost of creation of jobs in retailer r ∈ R
*scc*_*cc*_: cost of creation of job in collection centre cc ∈ CC
*srm*_*rm*_: cost of creation of jobs in remanufacturer rm ∈ RM
*src*_*rc*_: cost of creation of jobs in recycling centre rc ∈ RC
*cm*_*m*,*u*,p_: manufacturing cost of manufacturer m ∈ M for product u ∈ U in period p ∈ P
*cdc*_*dc*,*u*,*p*_: cost of distribution centre dc ∈ DC for product u ∈ U in period p ∈ P
*cr*_*r*,*u*,*p*_: cost of retailer r ∈ R for product u ∈ U in period p ∈ P
*ccc*_*cc*,*u*,*p*_: collection cost of collection centre cc∈ CC for product u ∈ U in period p ∈ P
*crm*_*rm*,*u*,*p*_: remanufacturing cost of remanufacturer rm ∈ RM for product u ∈ U in period p ∈P
*crc*_*rc*,*u*,*p*_: recycling cost of recycling centre rc ∈ RC for product u ∈ U in period p ∈ P
*tmdc*_*m*,*dc*,*p*_: transportation cost between manufacturer m ∈ M and distribution centre dc ∈ DC in period p ∈ P
*tdcr*_*dc*.*r*.*p*_: transportation cost between distribution centre dc ∈ DC and retailer r ∈ R in period p ∈ P
*trcc*_*r*,cc,*p*_: transportation cost between retailer r ∈ R and collection centre cc ∈ CC in period P ∈ P
*tccrm*_*cc*,*rm*,*p*_: transportation cost between collection centre cc ∈ CC and remanufacturing centre rm ∈ RM in period p ∈ P
*tccrc*_*cc*,*rc*,*p*_: transportation cost between collection centre cc ∈ CC and recycle centre rc ∈ RC in period p ∈ P
*trmdc*_*rm*,*dc*,*p*_: transportation cost between remanufacturing centre rm ∈ RM and retailer r ∈ R in period p ∈ P
*trmrc*_*rm*,*rc*,*p*_: transportation cost between remanufacturing centre rm ∈ RM and recycling centre rc ∈ RC in period p ∈ P
*α*_*u*,*p*_: percentage of return product from customer for product u ∈ U in period p ∈ P
*μ*_*u*,*p*_: percentage of return product from collection centre cc ∈ CC for product u ∈ U in period p ∈ P to recycle centre rc ∈ RC
*β*_*u*,*p*_: percentage of product sent to the distribution centre dc ∈ DC in period p ∈ P

Variables:

*mdc*_*m*,*dc*,*u*,*p*_: quantity of product u ∈ U shipped from manufacture m ∈ M to distribution centre dc ∈ DC in period p ∈ P
*dcr*_*dc*,*r*,*u*,*p*_: quantity of product u ∈ U shipped from distribution centre dc ∈ DC to retailer r ∈ R in period p ∈ P
*rcc*_r,*cc*,*p*,*u*_: quantity of product u ∈ U shipped from customer k ∈ K to collection centre cc ∈ CC in period p ∈ P
*ccrm*_*cc*,*rm*,*u*,*p*_: quantity of product u ∈ U shipped from collection centre cc ∈ CC to remanufacture centre rm ∈ RM in period p ∈ P
*ccrc*_*cc*,*rc*,*u*,*p*_: quantity of product u ∈ U shipped from collection centre cc ∈ CC to recycling centre rc ∈ RC in period p ∈ P
*rmdc*_*rm*,dc,*u*,*p*_: quantity of product u ∈ U shipped from remanufacture centre rm ∈ RM to retailer R ∈ R in period p ∈ P
*rmrc*_*rm*,*rc*,*u*,*p*_: quantity of product u ∈ U shipped from remanufacture centre rm ∈ RM to recycle centre rc ∈ RC in period p ∈ P
*δr*_r,*u*,*p*_: quantity of non-satisfied demand of retailer r ∈ R in period p ∈ P for product u ∈ U

Binary variables:

*bm*_*m*_: 1 if manufacturer m ∈ M is open; 0 otherwise
*bdc*_*dc*_: 1 if distribution canter dc ∈ DC is open; 0 otherwise
*br*_*r*_: 1 if retailer r ∈ R is open; 0 otherwise
*bcc*_*cc*_: 1 if collection canter cc ∈ CC is open; 0 otherwise
*brm*_*rm*_: 1 if remanufacturing canter rm ∈ RM is open; 0 otherwise
*brc*_*rc*_: 1 if recycling canter rc ∈ RC is open; 0 otherwise

#### Mathematical formulation

The mathematical formulation of the model is presented below. The model has two objectives: profit maximisation and job creation maximisation. Objective one is to maximise profit, which is calculated by deducting total cost from total revenue Eq (1). Total costs include cost of job creation Eq (2), fixed facility opening costs Eq (3), transportation cost Eq (4), processing cost Eq (5) and penalty cost of not satisfying demand Eq (6). Objective two is to maximise job creation by defining the number of jobs created with the opening of the facility Eq (7).

Objective Function Z1: maximize profit

$$\left(\sum_{r}\sum_{u}^{}\sum_{p}^{}(dr_{r,u,p}-\delta r_{r,u,p})*p_{u}\right)-$$ (1)

$$(\sum_{m}^{}\left((sm_{m}+om_{m})*bm_{m}\right)+\sum_{dc}^{}\left((sdc_{dc}+odc_{dc})*bdc_{dc}\right)+\sum_{r}^{}\left((sr_{r}+or_{r})*br_{r}\right)+\sum_{cc}^{}\left((scc_{cc}+occ_{cc})*bcc_{cc}\right)+$$

$$\sum_{rm}\left((srm_{rm}+orm_{rm})*brm_{rm}\right)+\sum_{}\left((src_{rc}+orc_{rc})*brc_{rc}\right)+$$ (2)

$$\overset{}{\underset{m}{\sum }}bm_{m}*om_{m}+\overset{}{\underset{dc}{\sum }}bdc_{dc}*odc_{dc}+\overset{}{\underset{r}{\sum }}br_{r}*or_{r}+\overset{}{\underset{cc}{\sum }}bcc_{cc}*occ_{cc}+\overset{}{\underset{rm}{\sum }}brm_{rm}*orm_{rm}+\overset{}{\underset{rc}{\sum }}brc_{rc}*orc_{rc}+$$ (3)

$$\sum_{m}^{}\sum_{dc}^{}\sum_{u}^{}\sum_{p}^{}mdc_{m,dc,u,p}*\text{tmd}{\text{c}}_{m,dc,p}+\sum_{dc}^{}\sum_{r}^{}\sum_{u}^{}\sum_{p}^{}dcr_{dc,r,u,p}*tdcr_{dc,r,p}+$$

$$\sum_{r}^{}\sum_{cc}^{}\sum_{u}^{}\sum_{p}^{}rcc_{\text{r},cc,u,p}*trcc_{\text{r},cc,p}+\sum_{cc}^{}\sum_{rm}^{}\sum_{u}^{}\sum_{p}^{}ccrm_{cc,rm,u,p}*tccrm_{cc,rm,p}+$$

$$\sum_{cc}^{}\sum_{rc}^{}\sum_{u}^{}\sum_{p}^{}ccrc_{cc,rc,u,p}*tccrc_{cc,rc,p}+\sum_{rm}^{}\sum_{dc}^{}\sum_{u}^{}\sum_{p}^{}rmdc_{rm,dc,u,p}*trmdc_{rm,dc,p}+$$

$$\sum_{rm}^{}\sum_{rc}^{}\sum_{u}^{}\sum_{p}^{}rmrc_{rm,rc,u,p}*trmrc_{rm,rc,p}+$$ (4)

$$\sum_{m}^{}\sum_{dc}^{}\sum_{u}^{}\sum_{p}^{}mdc_{m,dc,u,p}*cm_{m,u,p}+\sum_{dc}^{}\sum_{r}^{}\sum_{k}^{}\sum_{u}^{}\sum_{p}^{}dcr_{dc,r,u,p}*cdc_{dc,u,p}+$$

$$\sum_{cc}^{}\sum_{rm}^{}\sum_{rc}^{}\sum_{u}^{}\sum_{p}^{}(ccrm_{cc,rm,u,p}+ccrc_{cc,rc,u,p})*ccc_{cc,u,p}+$$

$$\sum_{rm}^{}\sum_{rc}^{}\sum_{dc}^{}\sum_{u}^{}\sum_{p}^{}(rmdc_{rm,\text{dc},u,p}+rmrc_{rm,rc,u,p})*crm_{rm,u,p}+$$ (5)

$$\sum_{r}^{}\sum_{u}^{}\sum_{p}^{}pr_{r,u,p}*\delta r_{r,u,p})$$ (6)

Objective Function Z2: Maximize job creation

$$\overset{}{\underset{m}{\sum }}jm_{m}*bm_{m}+\overset{}{\underset{dc}{\sum }}jdc_{dc}*bdc_{dc}+\overset{}{\underset{r}{\sum }}jr_{r}*br_{r}+\overset{}{\underset{cc}{\sum }}jcc_{cc}*bcc_{cc}+\overset{}{\underset{rm}{\sum }}jrm_{rm}*brm_{rm}+$$

$$\sum_{rc}^{}jrc_{rc}*brc_{rc}$$ (7)

Constraints:

The model constraints are defined as follows:

$$\sum_{dc}^{}mdc_{m,dc,u,p}\leq bm_{m}*km_{m}\;\forall m,u,p$$ (8)

$$\sum_{k}^{}mdc_{m,dc,u,p}+\sum_{r}^{}rmdc_{rm,dc,u,p}\leq bdc_{dc}*kdc_{dc}\;\forall dc,u,p$$ (9)

$$\sum_{dc}^{}dcr_{dc,r,u,p}\leq br_{r}*kr_{r}\;\forall r,u,p$$ (10)

$$\sum_{rm}^{}ccrm_{cc,rm,u,p}+\sum_{rc}^{}ccrc_{cc,rc,u,p}\leq bcc_{cc}*kcc_{cc}\;\forall cc,u,p$$ (11)

$$\sum_{r}^{}rmdc_{rm,dc,u,p}+\sum_{rc}^{}rmrc_{rm,rc,u,p}\leq brm_{rm}*krm_{rm}\;\forall rm,u,p$$ (12)

$$\sum_{rm}^{}rmrc_{rm,rc,u,p}+\sum_{cc}^{}ccrc_{cc,rc,u,p}\leq brc_{rc}*krc_{rc}\;\forall rc,u,p$$ (13)

$$\sum_{dc}^{}dcr_{dc,r,u,p}+\delta r_{r,u,p}\geq dr_{r,u,p}\;\forall r,u,p$$ (14)

$$\sum_{m}^{}mdc_{m,dc,u,p}+\sum_{k}^{}rmdc_{rm,dc,u,p}=\sum_{r}^{}dcr_{dc,r,u,p}\;\forall dc,u,p$$ (15)

$$\alpha_{u,p}*\sum_{dc}^{}dcr_{dc,r,u,p}=\sum_{cc}^{}rcc_{r,cc,u,p}\;\forall r,u,p$$ (16)

$$\mu_{u,p}*\sum_{r}^{}rcc_{r,cc,u,p}=\sum_{rc}^{}ccrc_{cc,rc,u,p}\;\forall cc,u,p$$ (17)

$$(1-\mu_{u,p})*\sum_{r}^{}rcc_{r,cc,u,p}=\sum_{rm}^{}ccrm_{cc,rm,u,p}\;\forall cc,u,p$$ (18)

$$\beta_{u,p}*\sum_{cc}^{}ccrm_{cc,rm,u,p}=\sum_{dc}^{}rmdc_{cc,dc,u,p}\;\forall rm,u,p$$ (19)

$$(1-\beta_{u,p})*\sum_{cc}^{}ccrm_{cc,rm,u,p}=\sum_{rc}^{}rmrc_{rm,rc,u,p}\;\forall rm,u,p$$ (20)

$$mdc_{m,dc,u,p},dcr_{dc,r,u,p},rcc_{r,cc,p,u},ccrm_{cc,rm,u,p},ccrc_{cc,rc,u,p},rmdc_{rm,dc,u,p},rmrc_{rm,rc,u,p}\geq 0$$ (21)

$$bm_{m},bdc_{dc},br_{r},bcc_{cc},brm_{rm},brc_{rc}\in \left\{0,1\right\}$$ (22)

Constraints (8)–(13) are capacity constraints. Constraint (8) ensures production capacity of manufacturing centre. Constraint (9) guarantees distribution centre has enough holding capacity for distribution of products. Constraint (10) shows retailers have enough capacity to respond to the demand. Constraint (11) expresses the capacity of collection centres for collecting return product. Constraints (12) and (13) ensure return products will not exceed the capacity of remanufacturing centres and recycling centres. Demand fulfilment is guaranteed by Constraint (14). Constraints (15)–(20) are equilibrium constraints, which ensure the flow balance at the distribution centre, retailer, collection centre, and remanufacturing centre. Constraint (21) imposes a non-negative decision variable. Finally, Constraint (22) characterises the binary variable.

### Non-dominated sorting genetic algorithm II (NSGA-II) solution to multi-objective problem

Here, a multi-objective model with two conflicting objectives was proposed. Multi-objective optimisation is to optimise two or more conflicting objectives with regard to a set of group constraints simultaneously [48]. If the optimisation of one objective leads to the automatic optimisation of the other, it is not a multi-objective optimisation. There is a group of solution methods for multi-objective problems called classical multi-objective optimisation methods such as *ε*–constraint, weighted sum, and goal programming. However, there are other evolutionary methods can be classified as non-classical method such as particle swarm optimization, simulated annealing, non-dominated sorting genetic algorithm-II (NSGA-II) and Strength Pareto Evolutionary Algorithm II. All these methods are Pareto-based techniques aiming to defines best Pareto optimal solution. Among these methods NSGA-II and Strength Pareto Evolutionary Algorithm II become common approach for solving multi-objective problems [49]. Deb et al. [50] expressed that the performance of NSGA-II in is much better in compared with other multi-objective optimizers. Fallah-Mehdipour et al. [51] demonstrated NSGA-II is more successful to provide optimum solution in comparison with multi-objective particle swarm optimization (MOPSO). In order to resolve the model, the non-dominated sorting genetic algorithm II (NSGA-II) is utilised.

Deb et al. [52] have indicate that any evolutionary algorithm which is applied to multi-objective problems should meet two criteria including set of solutions with Pareto frontier and uniform distribution of Pareto frontier. Therefore, the winner is the dominant solution and is selected for mating. Furthermore, if two solutions are non-dominant, the selected solution is the one with lower density of solution. “Solution density” is evaluated by means of a crowding distance. Moreover, the mutation randomly draws a solutions matrix. In fact, NSGA-II generates several solutions that, based on the criteria and constraints, select the feasible solution. This feasible solution will be pictured in the Pareto frontier figure.

NSGA-II algorithm starts with the generating of a random parent population with the size of n-Pop. By evaluating the population with the objective function, the population is ranked based on the non-domination sorting procedure to produce Pareto fronts. The algorithm gives the population a rank starting from level 1 for the best level, level 2 second best level and so on. The next step is calculation of “crowding distance” Eq (23) between members of each level [53]. In order to operate binary tournament selection, it is required to compute both the crowding distance and the rank for the members of the population. Two members of the population are selected and the one with larger crowding distance is selected if it is at the same level; otherwise, the member with the lower level is chosen. The next step consists of creating an offspring population and operation of cross-over and mutation. Finally, for the selection of the population with the same size of n-Pop by using sorting procedure, this process will stop when the stopping condition is met. At the end, a set of non-dominated Pareto-optimal solutions are attained which are the best in terms of multi-objective optimisation.

$$d_{I}=\sum_{i=1}^{B}\frac{f_{i}(x)-F}{f_{{}_{i}}^{\text{max}}-f_{i}^{\text{min}}}$$ (23)

## Results and discussions

In this section, in order to test the proposed model, an illustrative example is generated and the results are reported. It is found based on the literature review that several test problems have been proposed. Since, our proposed model is different from the others in the literature, the various problem sizes were randomly generated to test the proposed model. The detailed size of problems is shown in Table 2. For instance, in the test problem 1, there are four manufacturers, eight distribution centres, ten retailers, six collection centres, four remanufacturing centres and four recycling centres. Two types of products will be served to meet the customers’ demand in two particular periods.

**Table 2:** Size of test problem.

Problem No.	No. potential manufacturer	No. distribution centre	No. of retailer	No. of collection centre	No. of remanufacturing centre	No. of recycling centre	No. of period	No. of product
1	4	8	10	6	4	4	2	2
2	5	10	15	15	15	5	2	2
3	10	20	40	20	20	15	2	2
4	30	50	70	40	40	20	2	2

In the solution phase of the problem, the proposed model is determined with nominal data.

Table 3 shows the range of parameters that have been used in the model.

**Table 3:** Nominal data for proposed model.

Parameters	Corresponding random distribution
*om*_*m*_	Uniform (500000–700000)
*odc*_*dc*_, *occ*_*cc*_	Uniform (100000–200000)
*or*_*r*_	Uniform (50000–100000)
*orm*_*rm*_, *orc*_*rc*_	Uniform (200000–400000)
*dr*_r,*u*,*p*_	Uniform (300–500)
*pr*_r,*u*,*p*_	Uniform (40–90)
*km*_*m*_	Uniform (4500–5500)
*kdc*_*dc*_	Uniform (1800–2500)
*kr*_*r*_	Uniform (1300–2000)
*kcc*_*cc*_, *krm*_*rm*_, *krc*_*rc*_	Uniform (4000–8000)
*jm*_*m*_, *jdc*_*dc*_, *jr*_*r*_, *jcc*_*cc*_, *jrm*_*rm*_, *jrc*_*rc*_	Uniform (10–20)
*p*_*u*_	Uniform (5000–9000)
*sm*_*m*_, *sdc*_*dc*_, *sr*_*r*_, *scc*_*cc*_, *srm*_*rm*_, *src*_*rc*_	Uniform (400–600)
*cm*_*m*,*u*,p_, *cdc*_*dc*,*u*,*p*_, *ccc*_*cc*,*u*,*p*_, *crm*_*rm*,*u*,*p*_, *crc*_*rc*,*u*,*p*_	Uniform (3–8)
*tm dc*_*m*,*dc*,*p*_,	Uniform (2–4)
*tdcr*_*dc*.*r*.*p*_, *trcc*_*r*,cc,*p*_, *tccrm*_*cc*,*rm*,*p*_, *tccrc*_*cc*,*rc*,*p*_, *trmdc*_*rm*,*dc*,*p*_, *trmrc*_*rm*,*rc*,*p*_	Uniform (4–6)
*α*_*u*,*p*_	0.8
*μ*_*u*,*p*_	0.4
*β*_*u*,*p*_	0.8

The NSGA-II algorithm described in section 3.2 was programmed in MATLAB software, run on a personal computer with 2.27 GHz CPU and 4 GB main memory, and applied to solve the optimisation problem. The NSGA-II parameters were set as follows: population size 50; number of iteration 200; crossover rate 0.8; and mutation rate 0.5.

**Fig 2:**
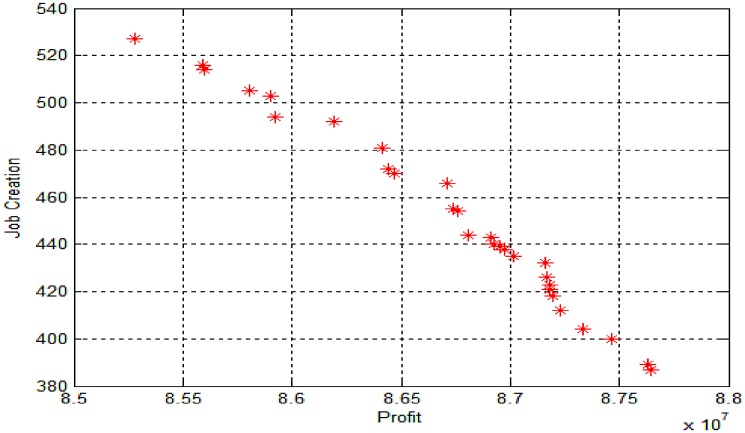
Pareto front solutions for proposed NSGA-II for test problem 1.

The result shown here in Fig 2 is the Pareto optimal solution. Hence, Pareto frontier optimal solutions graph is the key in multi-objective optimisation. The result for one random run shows 29 recognised particular solutions for the network configuration. Table 4 shows the list of solutions with objective value and network configurations. For instance, Table 4 shows solution number 2 states that manufacture centre 1, 3 and 4 need to be open; distribution centres 1, 2, 3, 5 and 6 should be open; collection centres 1 and 5 must be open; remanufacture and recycling centres 4 and 1 need to be open respectively.

**Table 4:** Optimal solutions for proposed NSGA-II for test problem 1.

Solution	Profit	Job creation	Manufacturer				Distribution centre								Collection centre						Remanufacturing centre				Recycling centre			
			1	2	3	4	1	2	3	4	5	6	7	8	1	2	3	4	5	6	1	2	3	4	1	2	3	4
1	85279482	527	*		*	*	*	*	*		*	*			*	*	*		*	*	*	*	*	*	*	*	*	*
2	87641846	387	*		*	*	*	*	*		*	*			*				*					*	*			
3	86416070	481	*		*	*	*	*	*		*	*			*	*	*	*	*	*		*		*	*	*		
4	86710477	466	*		*	*	*	*	*		*	*			*	*	*	*	*	*		*		*		*		
5	85923664	494	*		*	*	*	*	*		*	*			*	*			*	*	*	*		*	*	*	*	*
6	86194762	492	*		*	*	*	*	*		*	*			*	*	*	*	*	*	*			*	*	*		
7	87629902	389	*		*	*	*	*	*		*	*			*					*				*	*			
8	86465700	470	*		*	*	*	*	*		*	*			*	*	*	*	*	*	*			*		*		*
9	87231219	412	*		*	*	*	*	*		*	*					*			*	*	*			*			
10	85587892	516	*		*	*	*	*	*		*	*			*	*	*	*	*	*	*	*		*	*	*	*	*
11	86810434	444	*		*	*	*	*	*		*	*			*	*		*	*	*		*	*		*			
12	87466940	400	*		*	*	*	*	*		*	*			*		*		*					*	*			
13	87157776	432	*		*	*	*	*	*		*	*			*	*	*		*	*				*	*			
14	87017215	435	*		*	*	*	*	*		*	*				*	*		*	*		*		*	*			
15	87329595	404	*		*	*	*	*	*		*	*				*			*			*		*	*			
16	86757571	454	*		*	*	*	*	*		*	*			*	*	*	*	*	*	*			*	*			
17	86740537	455	*		*	*	*	*	*		*	*			*	*	*	*	*	*	*	*			*			
18	85807939	505	*		*	*	*	*	*		*	*			*	*	*	*	*	*		*		*	*	*	*	*
19	85905497	503	*		*	*	*	*	*		*	*			*	*	*	*	*	*	*	*		*	*	*		*
20	85596977	514	*		*	*	*	*	*		*	*			*	*	*	*	*	*	*	*	*	*	*	*		*
21	87196258	418	*		*	*	*	*	*		*	*			*		*		*			*		*	*			
22	86910446	443	*		*	*	*	*	*		*	*			*	*		*	*	*	*			*	*			
23	86441122	472	*		*	*	*	*	*		*	*			*	*	*	*	*	*	*			*		*	*	
24	87164673	426	*		*	*	*	*	*		*	*			*	*		*	*	*	*				*			
25	87182344	421	*		*	*	*	*	*		*	*			*	*				*		*		*	*			
26	87178143	423	*		*	*	*	*	*		*	*			*		*	*	*	*	*				*			
27	86925249	440	*		*	*	*	*	*		*	*			*		*	*	*	*	*			*	*			
28	86956071	439	*		*	*	*	*	*		*	*			*				*	*	*	*			*	*		
29	86970935	438	*		*	*	*	*	*		*	*			*				*	*	*			*	*	*		

### Fuzzy-based best compromise solution

Multi-object optimisation would not yield a single solution. Moreover, it has a conflicting goal in such a way that improvement in maximising one objective leads to sacrificing the other objective. Therefore, selecting one solution is a difficult task. However, we need to select one solution, the so-called ‘best compromise solution’, which to some extent satisfies other objectives.

**Fig 3:**
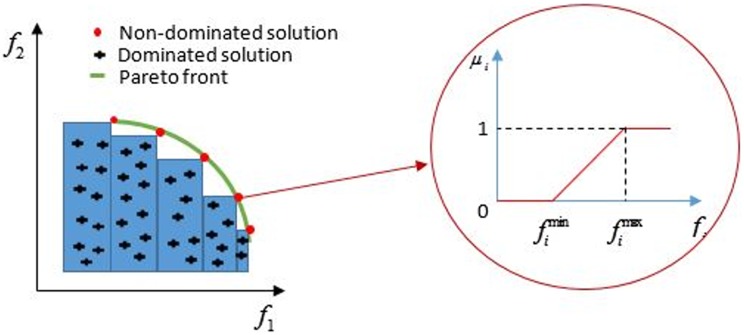
Fuzzy set mechanism (adopted from [54]).

After obtaining the Pareto optimal solution, the decision maker may need to choose one best solution based on his preference. To deal with this dilemma a fuzzy set mechanism (Fig 3) is defined to deal with this situation. A linear membership function *u*_*i*,*k*_ (*k*^th^ objective function, *i*^th^ solution) is defined Eq (24) for the objective function *F*_*i*_ as follows:

$$u_{i,k}=\{\begin{array}{ll} 1, & F_{i,k}\geq F_{k}^{\text{max}}\\ \frac{F_{i,k}-F_{k}^{\text{min}}}{F_{k}^{\text{max}}-F_{k}^{\text{min}}}, & F_{k}^{\text{min}}\leq F_{i,k}\leq F_{k}^{\text{max}}\\ 0, & F_{i,k}\leq F_{k}^{\text{min}} \end{array}$$ (24)

Where $F_{k}^{\text{min}}$ and $F_{k}^{\text{max}}$ are the value of the maximum and minimum of the objective function, respectively. Therefore, membership function indicates the degree of achievement of the original objective function as value 1 or 0 or in between. The normalised membership Function (25) for any non-dominated solution k is as follows:

$$u_{i}=\frac{\sum_{i=1}^{o}u_{i,k}}{\sum_{k=1}^{s}\sum_{i=1}^{o}u_{i,k}}$$ (25)

where *O* and *S* are the number of objective functions and non-dominated solutions, respectively. The solution with the maximum membership *u*_*k*_ can be seen as the best compromise solution.

The results show that the model in favour of job creation wants to open all the facilities to increase the number of jobs that have been created. However, the model, in favour of profit, wants to open a certain number of facilities to gain its maximum. The obtained Pareto-optimal fronts form the model depicted and are shown in Fig 2. According to the results, the best compromise solution (BCS) for this model is achieved at solution number 13. The BCS shows that manufacturers 1, 3, 4; distribution centres 1, 2, 3, 5, 6; collection centres 1, 2, 3, 5, 6; remanufacture centre 4 and recycle centre 1 should open. In this configuration, the profit of BCS is equal to 87157776 and job creation equals 432.

In order to test the validity of our model, several test problems have been chosen. Table 5 shows the result of one random run of the programme. As mentioned above the fuzzy best compromise optimum value of all four-test problems are also shown in Table 5. The result shows that the increase in the number of facilities will result in an increase in the number of jobs created with CLSC.

**Table 5:** List of solution for test problems.

No. of solutions	Test problem 1		Test problem 2		Test problem 3		Test problem 4	
	Profit	Job creation	Profit	Job creation	Profit	Job Creation	Profit	Job creation
1	85279482	527	119112809.3	876	312284744	1502	488440122	2719
2	87641846	387	132730299.3	490	329221184	1192	502803014.7	2037
3	86416070	481	122321036.7	873	329190519	1262	502183780.2	2203
4	86710477	466	120233008.7	874	321393206	1419	491621181.3	2713
5	85923664	494	132300367.9	513	324767450	1417	502382892.8	2074
6	86194762	492	125790850.4	859	318414951	1486	496838447.1	2483
7	87629902	389	131722642.9	618	328847354	1283	500965539.2	2217
8	86465700	470	132086397.1	553	316553242	1488	499377139.1	2345
9	87231219	412	131826653.6	586	328351219	1301	498679375.6	2359
10	85587892	516	124128043	862	319347964	1484	496616605.2	2537
11	86810434	444	126105275.5	847	327244060	1350	496137318.4	2549
12	87466940	400	127887250.5	774	325228509	1395	493770485.5	2626
13	87157776	432	131873379.1	562	320491619	1476	495253079	2555
14	87017215	435	126823467.2	825	326813591	1378	498495433.3	2379
15	87329595	404	132657783.3	498	321375670	1450	494341981.2	2608
16	86757571	454	128724210.5	770	313973499	1498	498403630.3	2404
17	86740537	455	127504709.2	799	315050442	1495	493216915.2	2661
18	85807939	505	130820443.1	676	328137445	1331	497279617.1	2477
19	85905497	503	129982484.1	722	327596204	1347	497776768.7	2422
20	85596977	514	130859080.1	658	326450379	1390	499525503.2	2318
21	87196258	418	126559400.1	838	315854282	1491	492612111.6	2668
22	86910446	443	129739865.9	735	328326154	1325	492393224.3	2704
23	86441122	472	127679852.4	786	320565230	1463	497842581.3	2405
24	87164673	426	127207713	803	321356257	1458	500265614.3	2261
25	87182344	421	129145792.3	764	325094551	1409	491687078.2	2709
26	87178143	423	130079179.2	716	314086770	1496	502495331.5	2057
27	86925249	440	126562256.1	832	326534008	1384	497555601.4	2440
28	86956071	439	130180584.3	707	325061299	1414	494551557	2591
29	86970935	438	131364398.6	636	324868976	1415	495049232.5	2590
30			130394377	696	326695263	1382	497404183	2460
31			127169137	812	326743040	1381	499967072.1	2308
32			130889517.8	650	314057791	1497	492580799.6	2679
33			128832834.8	768			496438378.2	2539
34			127740953.4	783			502607943.7	2042
35			129436446.4	744			492447927.7	2697
36			129658190.9	740			500644547	2220
37			130279978	702			500358958.9	2238
38			130527063.8	690			499636927.1	2309
39			130639880.6	682			500118068.5	2274
40			129354771.4	749			493640782	2642
41			131013434.6	644			495238143.5	2573
42			127169137	812			493415350.2	2652
43			131156664.6	643			500064442.5	2291
44			129214741.2	757			500632971.2	2233
45			131328701.6	639			497461307.9	2447
46			129262058.2	754			494466168	2601
47							495070854.5	2577
48							493522339	2645
49							500080396.8	2283
50							500394375.9	2236

### Managerial Insights

The goal of mathematical modelling is to model the real situation based on the mathematical formulation. In this way, the actual situation could be simulated in mathematical form to reduce cost and time. Mathematical programming can help the decision makers by providing a sort of “decisions alternative” with numerical information to give a better understanding of the actual situation. In our proposed CLSC network, decision makers want to know from the sort of potential facility locations, which one should be selected and the flow between each individual facility. For the decision maker, it is important to know the expected total cost and total profit of such a CLSC network. Moreover, it is important to know the number of employees required.

The result shows that the number of jobs created and profit are two conflicting objectives. Increase in one leads to decrease of the other one. Therefore, a decision maker wants to make a reasonable decision in this dilemma. This paper suggests a fuzzy best compromise solution to help the decision maker to cope with it. The result also shows that, with increase in size of network, job creation will increase. Increase in jobs is as result of the increase in number of facilities. Moreover, our proposed model provides strategic decisions for the decision maker to reduce unemployment rate based on the incentive which is gained from the policy makers, in such way that the decision maker builds the facilities in an area with high unemployment rate.

## Conclusions

As result of increased-environmental concern which has led firms to think about their environmental footprint, this study developed a CLSC network model to cope with conflicting environmental issues. Developing a proper network in the planning phase is, in fact, critical for an efficient and effective CLSC. Therefore, the study developed a mathematical model for end of life products. Moreover, the study considered the social responsibility of the firm in designing the CLSC network. Therefore, by proposing job creation as an objective targeted to reduce the unemployment rate, a multi-tier, multi-objective multi-product mixed integer linear programming system was developed to maximise profit and job creation. The result also shows that, with increase in size of network, job creation will increase. With fuzzy set theory, we defined a best solution from a range of feasible solutions. This paper opens the window for future research in designing CLSC in the perspective of corporate social responsibility.
