# Correction: Development of closed-loop supply chain network in terms of corporate social responsibility

**DOI:** 10.1371/journal.pone.0178723

**Published:** 2017-05-24

**Authors:** Ali Pedram, Payam Pedram, Nukman Bin Yusoff, Shahryar Sorooshian

The affiliation for the fourth author, Shahryar Sorooshian is incorrect. Affiliation **4** should be: Faculty of Industrial Management, Universiti Malaysia Pahang, Gambang Kuantan, Pahang, Malaysia.

The Acknowledgements section incorrectly spells the institution: Universiti Malaysia Pahang. The Acknowledgements section should read as follows: This research was supported by the University Malaya with Grant No. RP018B-13AET. The authors would like to thank the Centre of Manufacturing System Integration (MSI) and Centre for Product Design and Manufacturing (CPDM) of University Malaya for their support. The authors wish to express their gratitude to the Universiti Malaysia Pahang for funding for the publication (Grant No. RDU140107).
